# Identification of QTLs controlling aroma volatiles using a ‘Fortune’ x ‘Murcott’ (*Citrus reticulata*) population

**DOI:** 10.1186/s12864-017-4043-5

**Published:** 2017-08-22

**Authors:** Yuan Yu, Jinhe Bai, Chunxian Chen, Anne Plotto, Qibin Yu, Elizabeth A. Baldwin, Frederick G. Gmitter

**Affiliations:** 1 Citrus Research and Education Center, University of Florida, Lake Alfred, FL 33850 USA; 2 Horticultural Research Laboratory, ARS, USDA, Fort Pierce, FL 34945 USA; 3 Southeastern Fruit and Tree Nut Research Laboratory, ARS, USDA, Byron, GA 31008 USA

**Keywords:** Citrus, Aroma volatile, QTL, Molecular marker, Breeding

## Abstract

**Background:**

Flavor is an important attribute of mandarin (*Citrus reticulata* Blanco), but flavor improvement via conventional breeding is very challenging largely due to the complexity of the flavor components and traits. Many aroma associated volatiles of citrus fruit have been identified, which are directly related to flavor, but knowledge of genetic linkages and relevant genes for these volatiles, along with applicable markers potentially for expeditious and economical marker-assisted selection (MAS), is very limited. The objective of this project was to identify single nucleotide polymorphism (SNP) markers associated with these volatile traits.

**Result:**

Aroma volatiles were investigated in two mandarin parents (‘Fortune’ and ‘Murcott’) and their 116 F_1_ progeny using gas chromatography mass spectrometry in 2012 and 2013. A total of 148 volatiles were detected, including one acid, 12 alcohols, 20 aldehydes, 14 esters, one furan, three aromatic hydrocarbons, 16 ketones, one phenol, 27 sesquiterpenes, 15 monoterpenes, and 38 unknowns. A total of 206 quantitative trait loci (QTLs) were identified for 94 volatile compounds using genotyping data generated from a 1536-SNP Illumina GoldenGate assay. In detail, 25 of the QTLs were consistent over more than two harvest times. Forty-one QTLs were identified for 17 aroma active compounds that included 18 sesquiterpenes and were mapped onto four genomic regions. Fifty QTLs were for 14 monoterpenes and mapped onto five genomic regions. Candidate genes for some QTLs were also identified. A QTL interval for monoterpenes and sesquiterpenes on linkage group 2 contained four genes: geranyl diphosphate synthase 1, terpene synthase 3, terpene synthase 4, and terpene synthase 14.

**Conclusions:**

Some fruit aroma QTLs were identified and the candidate genes in the terpenoid biosynthetic pathway were found within the QTL intervals. These QTLs could lead to an efficient and feasible MAS approach to mandarin flavor improvement.

**Electronic supplementary material:**

The online version of this article (doi:10.1186/s12864-017-4043-5) contains supplementary material, which is available to authorized users.

## Background

Citrus is one of the most economically important fruit commodities in the world, including in the US. Citrus includes diverse fruit types preferably produced in adapted regions and climates. For example, sweet oranges (*Citrus sinensis* L. Osb.), mandarins (*C. reticulata* Blanco, commonly referred to as tangerines in the US), and grapefruit (*C. paradisi* Macf.) are three main types of citrus fruit produced in Florida, US. Next to orange, mandarin production worldwide is the second largest of all citrus fruit. Whereas sweet orange is primarily for juice processing, diverse mandarins are primarily for fresh consumption. Citrus fruit and juice are a well-known source of flavonoids, vitamin C, carotenoids, and volatile compounds which yield aromas and tastes specific to citrus fruits [1].

Citrus aroma is the result of a mixture of acids, alcohols, aldehydes, esters, ketones, monoterpenes, sesquiterpenes, and other volatiles [1–3]. The volatiles of sweet orange and grapefruit have been studied thoroughly because of their commercial importance in juice, and more than 300 volatiles have been reported in fresh orange juice [2]. For mandarins, several studies have been completed in peel oil and essence [4–7], and fresh fruits [8–16]. The aroma of mandarin juice is derived from a complex collection of several volatile compounds, rather than from any specific character-impact compounds [17]. In a review paper, a total of 37 consensus aroma volatiles were found in common across eight studies analyzing mandarins juice by gas chromatography mass spectrometry (GCMS), including seven alcohols, nine aldehydes, one ketone, 15 terpenes/hydrocarbons and five esters [3]. In addition, Miyazaki et al. [13, 14] identified a total of 146 volatiles in 25 mandarin hybrids and reported several new aroma active compounds by gas chromatography-olfactometry (GCO).

Flavor improvement is highly prioritized in recent mandarin breeding programs. The molecular markers linked to mandarin fruit aroma could facilitate genetic improvement and the development and release of new mandarin cultivars with improved fruit flavor. Compared to crop plants and vegetables, genetic mapping of fruit aroma in citrus, especially in mandarin, has not been performed. We recently constructed mandarin genetic linkage maps and identified quantitative trait loci (QTLs) for fruit quality characteristics [18]. In the present study, we investigated aroma volatiles in a mandarin F_1_ population using GCMS, and then we identified QTL regions controlling mandarin aroma volatiles. The long term objective is to understand characteristics of “good” mandarin fruit and to find fruit quality molecular markers for marker-assisted selection (MAS).

## Methods

### Plant materials

Mandarins (*Citrus reticulata* Blanco) ‘Fortune’, ‘Murcott’ and their 116 F_1_ hybrids, one tree each, were planted in 1991 for breeding purposes. Fruits were obtained from the University of Florida Citrus Research and Education Center groves in Lake Alfred, FL. Fruit of ‘Fortune’, ‘Murcott’ and F_1_ hybrids were harvested twice (a month apart) during their optimal commercial maturity in 2012 and 2013, respectively. The four harvests were presented as H1–12, H2–12, H1–13 and H2–13 in the main text subsequently.

### Sample preparation

Fruits were hand-harvested in the grove and transferred to the lab within 1–2 h for phenotypic evaluation. The phenotypic evaluation was performed as described [18]: 15 sound fruits from each tree were used to measure flavedo and juice color, fruit diameter (FD, in mm) and fruit weight (FW, in g) individually. Flavedo and juice color were measured using a Minolta CR-331 colorimeter. Color measurement score was described as Hunter color space value: *L* (black to white), *a* (green to red), *b* (blue to yellow), and *a* over *b* ratio. Then the 15 fruits were grouped into three pools with five fruits per pool, serving as three biological replicates. For the two harvests in 2012, the flavedo layer was first collected for another experiment and then the fruits were manually peeled and juiced (pressed) using a Hamilton Beach 932 manual commercial citrus juicer (Hamilton Beach, Southern Pines, NC, USA). For the two harvests in 2013, mandarin fruits were manually peeled and then juiced directly using the same procedure and juicer. Juice percentage (JP, in ml/100 g) was calculated, then color, soluble solids content (SSC, in g/100 mL), titratable acidity (TA, in g/100 mL, presented as percentage of citric acid) and volatiles were measured.

### Volatile extraction and GCMS analysis

A mixture of 3 mL of juice, 3 mL of saturated sodium chloride solution (359 g/L), and 6 μL of 1000 ppm 3-hexanone as internal standard were placed in a 20 ml glass vial and capped with a magnetic crimp cap with a silicone/PTFE septum (Gerstel, Linthicum, MD, USA). The vials were stored at −20 °C until analyzed. Frozen vials were thawed under tap water and loaded into the autosampler (Model MPS2, Gerstel Inc., Linthicum, MD) equipped with a cooled tray holder (Laird Tech, Sweden) controlled by a Peltier Thermostat (CTC Analytics AG, Switzerland). Samples were held 2–20 h at 4 °C until analyzed. For analysis, juice samples were incubated for 30 min at 40 °C. A 2.0 cm solid phase microextraction (SPME) fiber (50/30 μm DVB/Carboxen/PDMS; Supelco, Bellefonte, PA) was then exposed to the headspace for 30 min at 40 °C. After exposure, the SPME fiber was inserted into the injector of a GCMS (Model 6890, Agilent, Santa Clara, CA) to desorb the extract for 15 min at 250 °C. The GCMS equipment and settings were: DB-5 columns (60 m length, 0.25 mm i.d., 1.00 μm film thickness; J&W Scientific, Folsom, CA), coupled with a 5973 N MS detector (Agilent Technologies, Santa Clara, CA). The column oven was programmed to increase at 4 °C min^−1^ from the initial 40 °C to 230 °C, then ramped at 100 °C min^−1^ to 260 °C and held for 11.70 min for a total run time of 60 min. Helium was used as carrier gas at a flow rate of 1.5 mL min^−1^. Inlet, ionizing source and transfer line were kept at 250, 230, and 280 °C, respectively. Mass units were monitored from 30 to 250 m/z and ionized at 70 eV. Data were collected using the ChemStation G1701 AA data system (Hewlett-Packard, Palo Alto, CA). A mixture of C-5 to C-18 n-alkanes was run at the beginning of each day to calculate retention indices (RIs) [19].

### Volatile compound identification

Volatile compounds were identified by comparing their mass spectra with the Wiley HPCH 2205.L (Hoboken, NJ, USA), NIST05.L (NIST/EPA/NIH Mass Spectral Library; National Institute of Standards and Technology, Gaithersburg, MA, USA), and ADAMS.L [20] mass spectral database, as well as comparing RIs with published RIs on the same column. The amount of each aroma volatile was expressed as relative content (aroma volatile peak area over internal standard peak area).

### SNP genotyping, statistical analysis and QTL mapping

Mandarins were genotyped using an Illumina GoldenGate 1536-SNP array platform at the University of Florida’s Interdisciplinary Center for Biotechnology Research. The 1536 evenly distributed SNPs were initially mined from sweet orange bacterial artificial chromosome (BAC) end sequences [21], and have been applied to construct mandarin genetic linkage maps and identify fruit quality related QTLs using the same ‘Fortune’ x ‘Murcott’ population [18], and were then used for the QTL identification of mandarin juice volatiles in the published genetic linkage maps.

The descriptive statistical analysis of volatiles was performed using JMP Pro 11. T-test was applied to compare the means of each volatile between parents. In addition, Pearson correlation coefficients of each volatile for F_1_ hybrids were calculated. The analysis of variance (ANOVA) test was performed to test for differences of volatiles among the citrus selections. Due to non-normality, all volatile data were Log_10_-transformed and used in the subsequent statistical and QTL analysis. Principal component analysis (PCA) based on Pearson’s correlations was applied to differentiate tree individuals based on their volatile content using XLSTAT software (Addinsoft, Paris, France). Finally, the corresponding heat map representation of pairwise correlations was performed with JMP Pro 11. The QTL identification was performed on the published mandarin genetic linkage maps generated from the same population as described previously [18]. The non-transformed data were analyzed first by the Kruskal-Wallis rank-sum test using MapQTL 6.0, a significance level of *p* = 0.001 was used as threshold. Then Log_10_-transformed data were used in the subsequent QTL analysis using JMP Genomics 7 on the parental maps separately. QTLs were detected using composite interval mapping (CIM) [22, 23] with the expectation maximization (EM) mapping algorithm [24]. Logarithm (base) of odds (LOD) thresholds were calculated using a 1000-permutation test for each volatile on each map [25]. A QTL test step of 2 cM was used for CIM. QTLs were reported when their LOD values exceeded the LOD threshold, and they were literally named using volatile code followed by the number of the linkage group (LG) in which the QTL was located.

### Candidate gene screening

With colocation of SNPs on both genetic maps and the Clementine genome, the detected QTLs were compared to the map position of aroma volatile-related genes annotated in the citrus Clementine pathways from the CitrusCyc Pathways Database (http://pathways.citrusgenomedb.org, part of the Citrus Genome Database) to identify potential candidate genes. All the annotated genes related to the production of corresponding volatiles were considered in the candidate screening process, for example, valencene synthase (*TPS1*), geranyl diphosphate synthase 1 (*GPS1*), lipoxygenases (*LOX*), hydroperoxide lyase (*HPL*), hexenal isomerases (*HI*) and alcohol dehydrogenases (*ADH*). Candidate genes within a QTL/QTL cluster were searched only in the Clementine reference genome region delimited by the confidence interval of the QTL using the most proximal SNPs that were both on the Clementine genome sequence (http://www.phytozome.net/clementine.php) and the genetic linkage maps. Whether a gene was chosen as a candidate was based on the predicted annotation of the gene from the CitrusCyc Pathways Database, and was further confirmed by BLAST of the gene sequence from the Clementine genome against the Arabidopsis Information Resource (https://www.arabidopsis.org/).

## Results

### Volatile profiling and variation between ‘fortune’ and ‘Murcott’

More than 300 peaks were separated by GC in juice samples from ‘Fortune’, ‘Murcott’ and their hybrids over four harvests (H1–12, H2–12, H1–13 and H2–13). Of those, 148 volatiles were detected including one acid, 12 alcohols, 20 aldehydes, 14 esters, one furan, three aromatic hydrocarbons, 16 ketones, one phenol, 27 sesquiterpenes, 15 monoterpenes, and 38 unknown volatiles (Table 1, Additional files 1 and 2). Compared to ‘Murcott’, ‘Fortune’ produced a similar number of volatiles, but with lower concentration. The total relative content of volatiles in ‘Murcott’ was about 13 times as much as in ‘Fortune’ for both harvests in 2013, which was partly due to limonene. The ‘Murcott’ fruits contained about 20 times the amount limonene of ‘Fortune’. The most abundant volatiles identified in ‘Fortune’ included limonene (77.77%), valencene (2.63%), ethyl acetate (2.01%) and myrcene (1.53%); while ‘Murcott’ contained limonene (79.14%), hexanal (6.59%), myrcene (1.91%), heptanal (1.25%), and geranyl acetone (1.25%) in H1–13.

**Table 1:** Relative content of the 148 detected juice volatiles in ‘Fortune’ (For), ‘Murcott’ (Mur) and the F_1_ progeny in H1–13

Compound	Cluster^a^	For	Mur	F_1_	Compound	Cluster^a^	For	Mur	F_1_	Compound	Cluster^a^	For	Mur	F_1_
*Acid*					*Ketones*					*Furan*				
Nonanoic acid	B	nd	nd	nd-0.02	Acetone	A	tr^c^	0.02^c^	nd-0.05^f^	2-Pentylfuran	D	nd ^c^	0.04^c^	nd-0.02^e^
*Alcohols*					2-Butanone	D	nd	nd	nd-tr^e^	*Aromatic hydrocarbons*				
Ethanol^b^	A	0.01^c^	0.05^c^	nd-0.12	1-Penten-3-one	D	nd ^c^	0.01^c^	nd-0.03d					
2-Methyl-2-propanol					2,4-Pentanedione	D	nd	nd	nd-tr^e^					
	A	0.01	0.01	nd-0.25	4-Methyl-3-penten-2-one					α-Colocalene	D	nd	nd	nd-0.03
1-Octanol	D	tr^c^	nd^c^	nd-0.08		D	nd	nd	nd-0.02	Styrene	D	nd	nd	nd-tr
Linalool^b^	B	0.01^c^	0.06^c^	tr-0.97	4-Methyl-3-hexanone					1,3-Pentadiene	B	nd	nd	nd-0.03
(Z)-β-Terpineol	B	tr	nd	nd-0.18^d^		A	0.01^c^	nd^c^	nd-0.19					
Terpinen-4-ol	B	tr	0.01	nd-0.49	3-Heptanone	D	tr	nd	nd-tr^d^	*Phenol*				
α-Terpineol	B	tr^c^	0.03^c^	nd-0.81	1-Octen-3-one^b^	A	nd ^c^	0.02^c^	nd-0.07^d^	2,4-Di-tert-butylphenol				
(E)-Carveol^b^	B	nd	nd	nd-0.02	2,3-Octanedione	D	nd	nd	nd-0.04^e^		A	tr	nd	nd-0.04
(E)-Nerolidol	C	nd	nd	nd-0.12	6-Methyl-5-hepten-2-one					*Sesquiterpenes*				
Cubenol	C	nd	nd	nd-0.03^e^		A	nd ^c^	0.07^c^	nd-0.03^d^	α-Cubebene	C	nd	nd	nd-0.18^e^
Neointermedeol	D	nd	nd	nd-0.01	Dihydrocarvone	B	nd ^c^	0.05^c^	nd-0.03^d^	α-Copaene	C	tr^c^	tr^c^	nd-2.13^f^
Intermedeol	D	nd	nd	nd-0.01	(E)-Dihydro carvone					β-Elemene	C	nd	nd	nd-0.22^d^
*Aldehydes*						B	nd ^c^	0.02^c^	nd-0.03^d^	Caryophyllene	B	nd	nd	nd-0.13
Acetylaldehyde	A	tr^c^	tr^c^	nd-0.01	(D)-Carvone	B	tr^c^	0.11^c^	nd-0.15^f^	α-Guaiene	C	nd	nd	nd-0.09
Methacrolein	D	tr	nd	nd-tr^d^	Geranyl acetone	A	nd^c^	0.19^c^	nd-0.07^f^	β-Cubebene	C	nd	nd	nd-0.03
Pentanal	D	nd^c^	0.09^c^	nd-0.03^d^	β-Ionone^b^	D	nd	0.01	nd-0.01^d^	Aromadendrene	C	nd	nd	nd-0.12
Hexanal^b^	D	0.01^c^	1.02^c^	nd-0.35	Nootkatone^b^	D	nd	nd	nd-tr	Spirolepechinene	D	tr^c^	nd^c^	nd-0.01^e^
(E)-2-Hexenal	D	tr^c^	0.04^c^	nd-0.14	*Unknown*					RI1465	C	nd	nd	nd-0.15
Heptanal^b^	A	tr^c^	0.19^c^	nd-0.32	RI0547	C	tr	nd	nd-0.04^f^	RI1472	C	nd	nd	nd-0.09
(E)-2-Heptenal^b^	A	tr	nd	nd-0.19^f^	RI0754	A	tr	nd	nd-0.01^e^	α-Humulene	C	nd	nd	nd-0.24^d^
Octanal^b^	A	tr^c^	0.09^c^	nd-0.17	RI0941	A	0.01^c^	tr^c^	nd-0.11	(E)-Cadina-1(6),4-diene				
(E)-2-Octenal	A	tr^c^	0.05^c^	nd-0.22^f^	RI1005	A	tr^c^	nd^c^	nd-0.08^d^		C	nd	nd	nd-0.36
Nonanal	A	tr^c^	0.04^c^	nd-0.19	RI1028	D	tr^c^	0.04^c^	nd-1.25	γ-Muurolene	B	nd	nd	nd-0.2^e^
(E)-2-Nonenal^b^	A	tr^c^	0.03^c^	nd-0.1^d^	RI1076	B	nd	nd	nd-tr	α-Farnesene	B	nd	nd	nd-0.08
Decanal^b^	B	tr^c^	0.02^c^	nd-1.18	RI1087	A	tr^c^	nd^c^	nd-0.03^e^	Germacrene D	B	nd	nd	nd-0.04
p-Menth-1-en-9-al	B	nd	nd	nd-0.02^d^	RI1098	B	tr	nd	nd-0.13^d^	RI1495	D	tr^c^	nd^c^	nd-0.03^e^
β-Cyclocitral	B	nd ^c^	0.03^c^	nd-0.03^f^	RI1111	A	tr^c^	nd^c^	nd-0.04	Epizonarene	C	nd	nd	nd-0.22^e^
(E)-2-Decenal^b^	A	nd	nd	nd-0.05^e^	RI1128	A	tr^c^	nd^c^	nd-0.09	α-Muurolene	C	nd	nd	nd-0.49
Perillaldehyde	B	nd ^c^	0.01^c^	nd-2.61^f^	RI1155	B	nd	nd	nd-tr^f^	Valencene	D	0.03^c^	nd^c^	nd-0.43^d^
Undecanal	B	nd	nd	nd-0.22^e^	(+/−)-4-Acetyl-1-methylcyclohexene					α-Selinene	C	tr^c^	nd^c^	nd-0.26^d^
(E)-2-Undecenal	A	nd	nd	nd-0.04		D	nd^c^	0.01^c^	nd-tr^e^	Premnaspirodiene	D	nd	nd	nd-0.02^e^
Dodecanal^b^	B	nd	nd	nd-0.61^d^	RI1169	B	nd	tr	nd-0.07^f^	δ-Cadinene	C	tr^c^	nd^c^	nd-2.91^f^
β-Sinensal	C	nd	nd	nd-0.01	RI1195	B	nd	nd	nd-0.05	Calamenene	C	tr	nd	nd-0.37^d^
*Esters*					RI1211	D	nd	nd	nd-0.03^e^	7-epi-α-Selinene	D	tr^c^	nd^c^	nd-0.03^e^
Ethyl acetate	D	0.03	0.03	tr-1.55	RI1219	D	nd	nd	nd-0.02^e^	(E)-Cadina-1,4-diene				
Ethyl propanoate	D	nd	nd	nd-0.04^d^	RI1269	A	tr^c^	nd^c^	nd-0.05^d^		C	nd	nd	nd-0.34^e^
Ethyl 2-methylbutanoate^b^					RI1300	D	nd	nd	nd-0.01	α-Cadinene	C	nd	nd	nd-0.07
	C	nd	nd	nd-0.01^e^	RI1346	D	nd	nd	nd-0.07	α-Calacorene	C	nd	nd	nd-0.06
(Z)-3-Hexenyl acetate					RI1348	B	nd	nd	nd-0.23	*Monoterpenes*				
	D	nd	nd	nd-0.03^e^	RI1385	D	nd	nd	nd-0.04	α-Thujene	C	nd	nd	nd-0.03
Hexyl acetate	D	tr^c^	nd^c^	nd-tr^e^	RI1394	C	nd	nd	nd-0.14^e^	α-Pinene	B	0.01^c^	0.09^c^	tr-2.23
Octyl acetate	B	nd	nd	nd-3.61^d^	RI1398	B	nd	nd	nd-0.07	Myrcene^b^	B	0.02^c^	0.3^c^	tr-11.75
Nonyl acetate	B	nd	nd	nd-0.42^e^	RI1414	B	nd	nd	nd-1.05^e^	α-Phellandrene	B	tr	0.01	nd-0.7
(E)-Carvyl acetate	B	nd	nd	nd-0.19^e^	RI1420	B	nd	nd	nd-0.64^e^	α-Terpinene	B	tr^c^	0.02^c^	nd-1.71
Citronellyl acetate	B	nd	nd	nd-0.83^d^	RI1428	C	nd	nd	nd-0.02	p-Cymene^b^	D	0.01^c^	0.1^c^	nd-0.16
Neryl acetate	B	nd	nd	nd-0.26^e^	RI1483	D	nd	nd	nd-0.01	Limonene	B	0.97^c^	12.24^c^	0.12–98.92
Geranyl acetate	B	nd	nd	nd-0.1^e^	RI1540	C	nd	nd	nd-0.02^e^	β-Phellandrene^b^	B	tr^c^	0.04^c^	nd-0.77
Decyl acetate	B	nd ^c^	tr^c^	nd-1.29^e^	RI1549	C	nd	nd	nd-0.11	(E)-β-Ocimene	D	tr^c^	nd^c^	nd-0.06^d^
p-Menth-1-en-9-ol acetate					RI1554	D	nd	nd	nd-tr	γ-Terpinene^b^	B	tr	0.04	nd-0.97
	B	nd	nd	nd-1.2^d^	RI1557	B	nd	nd	nd-0.03^e^	Terpinolene^b^	B	0.01^c^	0.07^c^	nd-4.44
RI1561	C	nd	nd	nd-0.02	RI1595	D	nd	nd	nd-0.05	p-Mentha-2,4(8)-diene				
					RI1599	C	nd	nd	nd-0.04		A	0.01^c^	0.01^c^	nd-0.15
					RI1603	D	nd	nd	nd-0.08^e^	p-Mentha-1,3,8-triene				
					RI1606	D	nd	nd	nd-0.04^e^		B	nd^c^	tr^c^	nd-0.06^d^
					RI1609	B	nd	nd	nd-0.01	Allo-ocimene	B	nd	tr	nd-0.01
					RI1649	A	tr^c^	tr^c^	nd-0.05	Allo-ocimene isomer				
					RI1654	B	nd	nd	nd-0.01^e^		B	nd	nd	nd-0.02^d^

Data are normalized to the internal standard peak area. All values are mean of three biological replicates per genotype

tr. Peak was recognized but the value less than 0.0095

nd. Not detected

^a^ Clusters according to Fig. 3

^b^ Aroma active compounds reported by Miyazaki et al. [14]

^c^ Volatiles significantly different between parental lines ‘Fortune’ (FOR) and ‘Murcott’ (MUR) from the same harvest season (*p* < 0.05, Student’s t test)

^d^ Volatiles presented in 50% of the F_1_ hybrids (expected ratio 1:1, α =0.05, χ^2^ test)

^e^ Volatiles presented in 25% of the F_1_ hybrids (expected ratio 1:3, α =0.05, χ^2^ test)

^f^ Volatiles presented in 75% of the F_1_ hybrids (expected ratio 3:1, α =0.05, χ^2^ test)

**Fig. 1:**
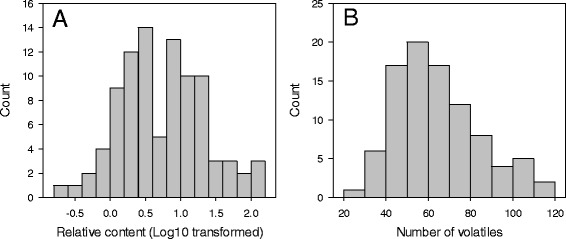
Frequency distributions of relative content and number of juice volatiles in ‘Fortune’ x ‘Murcott’ in H1–13. **a**) Relative content of volatiles, the value of relative content for each F_1_ hybrid was Log_10_ transformed, and then imported for analysis of distribution. **b**) Number of volatiles

The average number of volatiles detected in F_1_ hybrids was 63.7, and ranged from 28 to 111 (Table 1). The average relative content of volatiles detected in F_1_ hybrids was 14.4, and ranged from 0.24 to 133.24. Three volatiles were present in all F_1_ hybrids, including linalool, myrcene and limonene; and another 18 volatiles were detected in 90–99% of all F_1_ hybrids (Additional file 1). On the other hand, 19 compounds were detected in less than 10% of all hybrids. Among the 148 detected volatiles, 64 volatile compounds were detected specifically in F_1_ hybrids but were not present in either of the parents. The total content of volatiles and number of volatiles in F_1_ progeny showed continual variation, although some of their distributions were generally skewed and/or significantly deviated from normality (*p* = 0.05) based on the Shapiro-Wilk test [26] (Fig. 1, Additional file 3). For instance, the total content displayed a distribution significantly deviated from normality in H1–12, so did the number of volatiles in H1–12, H1–13 and H2–13. Transgressive segregations were found in both directions where decanal, perillaldehyde, ethyl acetate, octyl acetate, decyl acetate, p-menth-1-en-9-ol acetate, α-pinene, myrcene, limonene and terpinolene exhibited the most significant transgressions (Table 1, Additional file 2).

A total of 20 volatile compounds were reported with odor activities in the F_1_ individual FoMu-081 [14]. In the present study (Table 1, Additional file 2), (E)-2-decenal described as minty/piney, 1-octen-3-one described as mushroom and β-ionone described as floral, were found in ‘Murcott’, but not in ‘Fortune’ in 2013. Four aroma active compounds were detected only in F_1_ hybrids in 2013, including (E)-carveol described as minty, dodecanal described as green, ethyl 2-methylbutanoate described as fruity and nootkatone described as spicy/woody. Two aroma active compounds were present in all F_1_ hybrids in 2013, including linalool described as floral and myrcene described as metallic.

**Fig. 2:**
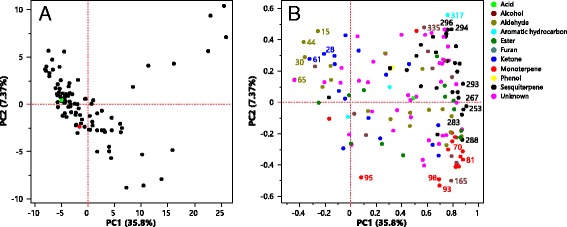
Principle component analysis (PCA) of 148 volatile compounds determined in ‘Fortune’, ‘Murcott’ and the 116 F_1_ hybrits in H1–13. **a**) Points show the PCA scores of each line, ‘Fortune’ and ‘Murcott’ are highlighted in green and red, respectively. **b**) Loading plots of each volatile compound. The numbers can be found in the second column (volatile code) preceded by Pk in Additional files 1 and 2

Principal component analysis (PCA) was employed to analyze the differences in volatile composition in ‘Fortune’ x ‘Murcott’ within each harvest, and the results from H1-13 were shown here as a representative (Fig. 2). The first and second principal components (PC1 and PC2) represented 35.8 and 7.37% of the total variance. The parents were relatively well separated from each other, but not differentiated from most of their F_1_ progeny, indicating that ‘Fortune’ and ‘Murcott’ exhibited relatively different volatile compositions. F_1_ individuals formed a distribution trend from top left, with more of the compounds like hexanal (44, volatile code as shown in Additional file 2), methacrolein (15), pentanal (30), 3-heptanone (61), 1-penten-3-one (28), heptanal (65), and to the bottom right, with more of the volatiles like α-terpineol (165), β-phellandrene (98), α-phellandrene (93), p-cymene (95). Some hybrids on the top right contained much more volatiles compared to others, like F_1_ individuals FoMu-115, FoMu-020, FoMu-091 and FoMu-076 with 111, 109, 101 and 108 volatiles respectively. Principal component, PC1, separated the individual hybrids primarily based on the relative content of α-humulene (267), δ-cadinene (288), (E)-cadina-1,4-diene (293), caryophyllene (253), α-muurolene (283), myrcene (81) and α-pinene (70), while α-phellandrene (93), p-cymene (95), β-phellandrene (98), α-colocalene (317), α-calacorene (296), α-cadinene (294), α-terpineol (165) and neointermedeol (335) were important for the separation of mandarin hybrids across PC2.

### Correlation analysis of volatiles in ‘Fortune’ x ‘Murcott’

The pair-wise correlation coefficient for each volatile against every other compound was calculated in order to identify co-regulated compounds. Pearson correlation was performed for F_1_ progeny juice samples in H1–13. Of the 11,476 pairs analyzed, 5885 resulted in significant correlations (*p* < 0.05) (data not shown). Of these 5885 pairs, most of them (5724) exhibited positive correlation coefficients. The strongest positive correlations were found between α-muurolene with δ-cadinene (*r* = 0.999) and (E)-cadina-1,4-diene (*r* = 0.998). Strong positive (*r* > 0.5) correlations were also found between (E)-2-octenal with (E)-2-nonenal (*r* = 0.997) and heptanal (*r* = 0.996), between δ-cadinene and (E)-cadina-1,4-diene (*r* = 0.996), between nonyl acetate with octyl acetate (*r* = 0.995) and decyl acetate (*r* = 0.994). The strongest negative correlations were observed between 3-heptanone with linalool (*r* = −0.26), and limonene (*r* = −0.25) with dihydrocarvone (*r* = −0.24).

The 148 volatile compounds were categorized into 12 groups based on their chemical structure including acid, alcohol, aldehyde, ester, ether, furan, aromatic hydrocarbon, ketone, phenol, sesquiterpene, monoterpene and unknown [13]. The pair-wise correlation coefficient for each chemical group against every other group was calculated for juice samples from hybrids (Additional file 4). Strong positive correlations were observed for most of the major groups over four harvests, except for the ketone group which showed poor or no correlations. The ketone group was correlated with alcohols (*r* = 0.49 and 0.56) in 2013, but showed no strong correlations with any other groups.

The pair-wise correlation coefficient for each chemical group against every fruit characteristic was also calculated for hybrids (Additional file 5), and few strong correlations (|r| > 0.5) were observed. In 2013, total volatile content positively correlated with the traits TA and juice color space value *L*, but negatively correlated with fruit diameter, fruit weight, juice percentage, SSC/TA ratio, flavedo and juice color space value *a* and *a*/*b* ratio. Significant (*p* < 0.05) positive correlations were found between TA with alcohols, aldehydes, esters and monoterpenes over two harvests in 2013. Juice color space value *a* was negatively correlated with monoterpenes over four harvests, and juice color space value *b* displayed relatively strong correlations with alcohols, aldehydes, esters and monoterpene in H1–13. The pair-wise correlation coefficient for color characteristic against every carotenoid-derived volatile including 6-methyl-5-hepten-2-one, β-cyclocitral, isophorone, neryl acetate, geranyl acetone and β–ionone, was calculated for hybrids (Additional file 6). Only a few stable significant correlations were found. Flavedo and juice color space value *a* and *a*/*b* ratio displayed stable significant negative correlations with β-cyclocitral and neryl acetate, and a positive correlation with geranyl acetone over two or more harvests.

### Cluster validation using correlation analysis

HCA (hierarchical cluster analysis) based on pairwise correlation coefficients were run individually for four harvests, and four distinct groups of volatiles were formed in H1–13 (Additional file 7). Cluster A included 26 volatiles, of which the majority were aldehydes, ketones and unknown compounds. Cluster B contained two sub-clusters B1 and B2 with 24 and 27 volatiles respectively. The B1 sub-cluster consisted of mainly aldehydes, esters, monoterpenes and unknown compounds, and the B2 comprised mainly alcohols, sesquiterpenes and monoterpenes. Cluster C included two sub-clusters C1 and C2 with 18 and 12 volatile compounds respectively. The C1 sub-cluster contained mainly sesquiterpenes, and the C2 comprised mainly sesquiterpenes and unknown compounds. Cluster D included two sub-clusters D1 and D2 with 20 and 23 volatiles respectively. The D1 consisted of mainly sesquiterpenes and unknown compounds, and the D2 contained mainly aldehydes, esters and ketones.

### Genetic mapping of QTLs controlling aroma compounds in ‘Fortune’ X ‘Murcott’

**Fig. 3:**
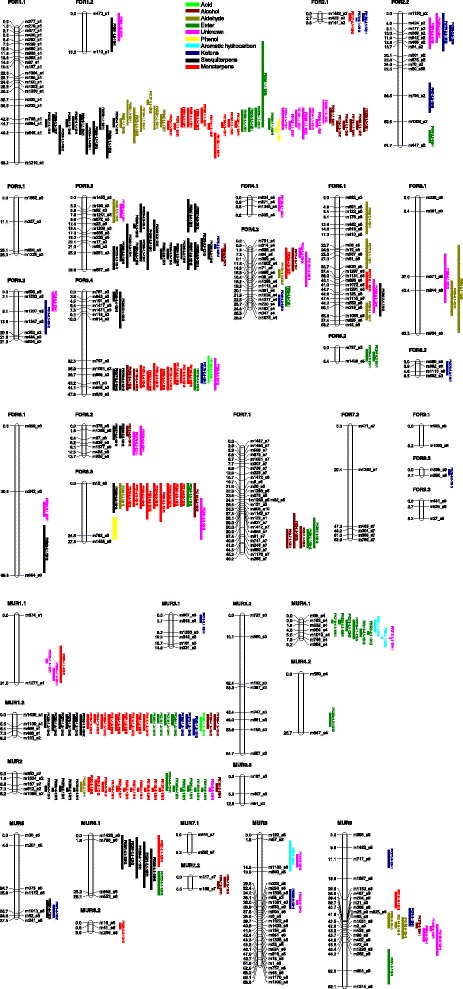
Parental linkage groups with QTLs associated with mandarin juice volatiles. QTLs are identified on the ‘Fortune’ (For) and ‘Murcott’ (Mur) linkage groups. Color bars represent QTLs with 1-LOD support intervals. QTLs are literally named using trait abbreviation followed by the number of the LG in which the QTL is located. The details of QTLs are presented in Additional file 8

Some volatiles displayed qualitative variations, which suggested single locus inheritance. Pentanal, valencene and α-selinene were detected in juice samples from one parental line and the segregation pattern in the progeny matched a 1:1 ratio (α =0.05, χ^2^ test), while (E)-2-octenal, acetone and (D)-carvone were present in both parents and the segregation pattern matched a 3:1 ratio (Additional file 2). In addition, 12 volatile compounds were absent in parental lines and the segregation pattern matched a 3:1 ratio. All QTLs for volatile compounds were identified using Log_10_ transformed data. A total of 244 significant associations were found between molecular markers and 94 volatiles (Fig. 3 and Additional file 8). QTLs for each volatile located in the same chromosomal regions from different harvest seasons were considered to be the same, hence the 244 associations then were summarized into 206 QTLs, with 25 (12.14%) being repeatable (Table 2). Within the 25 repeatable QTLs, two, two, five, nine and five were detected for alcohols, aldehydes, esters, sesquiterpenes and monoterpenes, respectively. Out of the 206 QTLs, 41 QTLs were identified for 17 aroma active compounds. The number of QTLs ranged from zero on linkage groups FOR3.1, FOR7.2, FOR9.1, FOR9.3, MUR3.2, MUR3.3 and MUR7.1 to 47 on FOR1.1. The average R^2^, explaining phenotypic variance, for all volatiles was 21.9%, and ranged from 14.49% for nonanoic acid to 59.82% for valencene in H1–13 with the highest LOD value of 17.22.

**Table 2:** Repeatable QTLs detected for juice volatiles in the mandarin F_1_ population, ‘Fortune’ x ‘Murcott’, using Kruskal-Wallis (K-W) and Composite Interval Mapping (CIM)

Compound	QTL^a^	Harvest date	Linkage group	K-W^b^	LOD Thr^c^	LOD max^d^	Position^e^	Nearest marker	Marker position^f^	Effect	R2 (%)^g^
Ethanol	*PK3–7.1*	H1–12	FOR7.1	******	3.07	4.58	40.592	m741_s7	40.592	−0.61	23.45
		H2–12	FOR7.1	******	2.91	3.65	40.592	m741_s7	40.592	−0.63	22.46
		H2–13	FOR7.1	*****	2.77	3.22	40.592	m741_s7	40.592	−0.51	17.72
	*PK3–7.2*	H2–12	MUR7.2	*	2.83	7.05	4	m906_s7	6.4	2.29	38.86
		H1–13	MUR7.2	*	2.54	5.49	4	m906_s7	6.4	1.28	25.49
Heptanal	*PK65–1.1*	H1–13	FOR1.1	-	2.87	3.54	42.5	m783_s1	42.9	−0.72	17.1
		H2–13	FOR1.1	*****	2.81	3.37	44.7	m884_s1	44.7	−0.83	18.49
Octanal	*PK90–8.1*	H1–12	FOR8.1	****	2.94	3.19	43.4	m244_s8	43.4	−0.67	16.96
		H2–13	FOR8.1	***	2.88	3.62	49.4	m244_s8	43.4	−0.59	19.69
Ethyl acetate	*PK20–7.1*	H2–12	FOR7.1	*******	2.83	3.95	40.592	m741_s7	40.592	−0.71	24.11
		H2–13	FOR7.1	******	2.86	3.6	43.559	m860_s7	44.264	−0.72	19.58
Citronellyl acetate	*PK216–1.2*	H1–13	MUR1.2	-	2.68	3.35	0	m1436_s1	0	0.97	16.4
		H2–13	MUR1.2	*******	2.76	3.94	0	m1436_s1	0	1.24	21.5
	*PK216–4.1*	H1–12	MUR4.1	*******	2.54	7.37	0.91	m163_s4	0.91	0.67	34.93
		H2–12	MUR4.1	*******	2.65	4.49	4.55	m959_s4	4.55	0.66	26.89
		H2–13	MUR4.1	****	2.76	3.02	5.46	m1019_s4	5.46	0.95	16.93
Neryl acetate	*PK220–4.1*	H1–12	MUR4.1	*******	2.68	7.93	0.91	m163_s4	0.91	1.06	37
		H2–12	MUR4.1	*******	2.67	6.76	4.55	m959_s4	4.55	1.07	37.59
		H2–13	MUR4.1	*******	2.98	8.35	5.46	m1019_s4	5.46	2.64	40.1
p-Menth-1-en-9-ol acetate	*PK244–2*	H1–12	MUR2	******	2.5	4.06	5.46	m167_s2	5.46	−0.53	21.06
		H1–13	MUR2	*****	2.63	4.18	7.28	m512_s2	7.28	−1.01	20.07
1,3-Pentadiene	*PK9–4.1*	H2–12	MUR4.1	*******	2.83	6.59	8.19	m358_s4	8.19	1.12	36.86
		H2–13	MUR4.1	******	2.56	3.49	0	m65_s4	0	0.64	19.3
RI1549	*PK300–6.2*	H1–12	FOR6.2	*****	2.87	3.49	12.75	m485_s6	12.75	−0.86	18.39
		H1–13	FOR6.2	****	2.69	3.27	1.82	m1258_s6	1.82	−0.52	15.91
Spirolepechinene	*PK262–3.3*	H2–12	FOR3.3	*******	2.9	7.98	25.9	m261_s3	25.9	−1.63	42.68
		H1–13	FOR3.3	*******	3.01	3.46	25.9	m261_s3	25.9	−0.41	16.73
	*PK262–3.4*	H1–12	FOR3.4	******	2.8	3.32	0	m751_s3	0	0.89	17.58
		H2–13	FOR3.4	*******	2.79	4.37	3.64	m643_s3	3.64	0.68	23.29
RI1495	*PK280–3.3*	H1–12	FOR3.3	*******	2.98	6.67	25.9	m261_s3	25.9	−1.18	32.23
		H2–12	FOR3.3	*******	2.91	12.53	12.503	m572_s3	9.645	−2.04	58.3
		H1–13	FOR3.3	*******	2.95	5.88	6.824	m92_s3	6.824	−0.7	26.76
		H2–13	FOR3.3	*******	3.04	10.98	25.9	m261_s3	25.9	−1.31	48.6
Valencene	*PK284–3.3*	H1–12	FOR3.3	****	2.81	3.51	23.042	m66_s3	21.042	−0.62	18.5
		H2–12	FOR3.3	*******	2.87	9.4	21.042	m66_s3	21.042	−0.97	48.09
		H1–13	FOR3.3	*******	2.95	17.22	25.9	m261_s3	25.9	−1.83	59.82
		H2–13	FOR3.3	*******	2.96	12.72	25.9	m261_s3	25.9	−3.26	53.74
α-Selinene	*PK286–3.3*	H1–13	FOR3.3	*******	2.86	4.74	25.9	m261_s3	25.9	−0.85	22.21
		H2–13	FOR3.3	*******	2.91	11.28	25.042	m261_s3	25.9	−1.57	49.51
Premnaspirodiene	*PK287–3.3*	H2–12	FOR3.3	******	3.05	4.09	23.042	m66_s3	21.042	−1.3	24.83
		H1–13	FOR3.3	*******	2.99	3.73	5.889	m156_s3	5.889	−0.49	17.92
		H2–13	FOR3.3	*******	3	5.67	7.758	m1241_s3	7.758	−0.84	29.08
δ-Cadinene	*PK288–6.1*	H2–12	MUR6.1	****	2.69	6.82	7.82	m796_s6	1.82	0.94	37.84
		H1–13	MUR6.1	*****	2.6	3.36	9.82	m796_s6	1.82	0.98	16.48
		H2–13	MUR6.1	**	2.62	2.96	15.82	m858_s6	25.21	1.18	16.61
Calamenene	*PK291–6.1*	H2–12	MUR6.1	***	2.67	4.96	5.82	m796_s6	1.82	0.61	29.25
		H1–13	MUR6.1	***	2.49	3.14	11.82	m796_s6	1.82	0.88	15.47
7-epi-α-Selinene	*PK292–3.3*	H1–12	FOR3.3	******	2.99	4.54	21.042	m66_s3	21.042	−1.07	23.27
		H2–12	FOR3.3	*******	2.99	11.46	20.108	m17_s3	20.108	−1.89	55.05
		H2–13	FOR3.3	*******	3.04	12.2	25.042	m261_s3	25.9	−1.23	52.25
α-Terpinene	*PK94–2*	H1–13	MUR2	*******	2.63	4.5	8.2	m1069_s2	8.2	−0.66	21.43
		H2–13	MUR2	***	2.67	3.09	7.28	m512_s2	7.28	−0.58	17.29
β-Phellandrene	*PK98–1.1*	H1–13	FOR1.1	*******	2.92	5.81	44.7	m884_s1	44.7	1.12	26.48
		H2–13	MUR1.1	***	2.72	3.43	30.95	m1271_s1	30.95	−0.81	18.98
	*PK98–2*	H1–13	MUR2	******	2.56	4.45	8.2	m1069_s2	8.2	−0.73	21.22
		H2–13	MUR2	***	2.68	2.98	7.28	m512_s2	7.28	−0.83	16.74
γ-Terpinene	*PK106–6.3*	H1–13	FOR6.3	***	2.9	3.4	0	m12_s6	0	0.61	16.49
		H2–13	FOR6.3	****	2.88	4.46	0	m12_s6	0	1.01	23.66
Allo-ocimene isomer	*PK136–1.1*	H1–13	FOR1.1	*******	2.73	8.42	44.7	m884_s1	44.7	1.31	35.97
		H2–13	FOR1.1	***	2.82	4.09	44.7	m884_s1	44.7	0.98	21.97

^a^ QTLs are literally named using compound code followed by the number of the linkage group in which the QTL is located

^b^ Significance level of Kruskal-Wallis test. - *p* > 0.05; * *p* < 0.05; ** *p* < 0.01; *** *p* < 0.001; **** *p* < 0.0001; ***** *p* < 0.00001; ****** *p* < 0.000001; ******* *p* < 0.0000001

^c^ LOD threshold determined by 1000 permutation tests for each trait in each harvest and each map

^d^ The LOD maximum for each QTL

^e^ The QTL position (in cM) from the top of LG

^f^ The nearest marker position (in cM) from the top of LG

^g^ The percentage of the total phenotypic variation explained by the QTL

QTLs were grouped into four and three main clusters in the FOR and MUR maps, respectively. The first cluster on the FOR map was located on FOR1.1, where QTLs were identified for 47 volatiles including six alcohols, six aldehydes, five esters, one phenol, 11 sesquiterpenes, 10 monoterpenes and eight unknown compounds. Out of the 47 volatiles, nine volatiles were aroma active compounds, including linalool, heptanal, (E)-2-heptenal, decanal, dodecanal, myrcene, β-phellandrene, γ-terpinene and terpinolene. The second and third clusters were both located on FOR3.3. The second cluster was made up of nine QTLs identified for one alcohol, one ketone and seven sesquiterpenes. The R^2^ was 59.82% for valencene in H1–13. Within the confidence intervals of the cluster, no candidate genes were found related to terpenoid regulation, even though the gene encoding valencene synthase (TPS1) was located on the Clementine genome scaffold 3. The third cluster of QTLs was identified for one acid, one alcohol, one ester, one ketone, two sesquiterpenes, eight monoterpenes and one unknown compound. The fourth cluster was located on FOR6.3, included QTLs for one alcohol, one aldehyde, one ester, one sesquiterpene and eight monoterpenes. Five of the eight monoterpenes were aroma active compounds including linalool, myrcene, terpinolene, β-phellandrene and γ-terpinene.

The first cluster on the MUR map was located on MUR1.2, and contained QTLs for 24 volatiles including one acid, two alcohols, three esters, three ketones, six sesquiterpenes and nine monoterpenes. The second cluster was located on MUR2, consisted of QTLs for 22 volatiles including one alcohol, one aldehyde, five esters, five sesquiterpenes, nine monoterpenes and one unknown compound. Four of them were aroma active compounds including myrcene, β-phellandrene, γ-terpinene and dodecanal. Within the confidence intervals of the QTL cluster on MUR2, 572 annotated genes were listed. After screening, four of these genes, *GPS1*, *TPS3*, *TPS4* and *TPS14*, encoding geranyl diphosphate synthase 1, terpene synthase 3, terpene synthase 4 and terpene synthase 14 respectively, were probably related to terpenoid regulation based on their biological function as studied in model plant species such as *Arabidopsis* (Additional file 9). The third cluster was located on MUR4.1, where QTLs were identified for two esters, one aromatic hydrocarbon and one unknown compound.

Repeatable and overlapped QTLs were also detected elsewhere for biologically related compounds in FOR and MUR maps. Overlapped QTLs were identified for (D)-carvone and 6-methyl-5-hepten-2-one on FOR2.1, (E)-dihydro carvone and (D)-carvone on FOR2.2, nonyl acetate and p-menth-1-en-9-ol acetate on FOR5.2, and α-copaene and δ-cadinene on FOR6.2. Repeatable QTLs were overlapped for δ-cadinene and calamenene on MUR6.1, and ethanol and ethyl acetate on FOR7.1. Eight QTLs on MUR9 were overlapped for one alcohol, three aldehydes, one ketone and three unknown compounds, and three of them were aroma active compounds including (E)-carveol, (E)-2-nonenal and (E)-2-decenal.

## Discussion

### Mandarin juice aroma volatile profiles

During the sample preparation, any scraping of the albedo or squeezing of the flavedo may contaminate juice samples with peel components and change the aroma volatile profiles due to high concentration of volatile compounds in mandarin peel oil. In this study, mandarin fruits were manually peeled and juiced using a juicer. For the two harvests in 2012, the flavedo layer was first collected for another experiment and then the fruits were peeled. In order to minimize the error and exhibit the truest mandarin juice volatile composition, only aroma volatiles presented in all four harvests were reported here, and the QTLs only identified in 2012 were discarded. In this study, 148 volatiles were detected by GCMS in juice samples prepared from manually peeled fruits of ‘Fortune’, ‘Murcott’ and their 116 F_1_ hybrids. A total of 55 and 48 volatiles were detected for ‘Fortune’ and ‘Murcott’ respectively in 2013. Moshonas & Shaw [27] detected and quantified 42 juice volatile compounds from unpeeled fruits of 15 mandarin selections, and 34 volatiles from ‘Murcott’. In juice samples prepared from unpeeled fruits, 82 and 80 volatiles were reported from ‘Murcott’ and one hybrid of ‘Fortune’ x ‘Murcott’ respectively [13]. In the present study, the number of volatiles detected in F_1_ hybrids ranged from 28 to 111 volatiles with average number of 63.7 in H1–13. Kerbiriou et al. [10] studied aroma volatiles in juice samples extracted from unpeeled fruits of 56 mandarin hybrids and detected 225 volatile compounds, ranging from 21 to 86 volatiles, and the subsequent study of 25 genetically related mandarin materials identified 146 juice aroma volatiles from unpeeled fruits, and the range for each sample was 52 to 118, with an average number of 77 per sample [13].

Limonene, ethanol, α-pinene and cymene presented in all juice samples from 56 mandarin hybrids [10], while Miyazaki et al. [13] found 15 consensus aroma volatiles from 25 mandarin hybrids. Tietel et al. [3] identified 37 consensus aroma volatiles in mandarin juice present in at least four previous studies and considered nine volatile compounds as core aroma volatiles including linalool, α-terpineol, terpinen-4-ol, nonanal, decanal, carvone, limonene, α-pinene and myrcene. Here, limonene, myrcene and linalool were present in all F_1_ hybrids of ‘Fortune’ x ‘Murcott’, and another 18 volatile compounds were detected in 90–99% of all F_1_ hybrids, such as α-pinene, ethanol, hexanal, α-phellandrene and terpinolene (Additional file 1). Conversely, 19 volatiles were detected in less than 10% of all F_1_ hybrids and may be considered as hybrid specific volatile compounds, such as (E)-carveol and nootkatone presented in only nine hybrids.

‘Fortune’ was reported to be derived from a ‘Clementine’ x ‘Orlando’ tangelo [28], while ‘Murcott’ was reported as a mandarin sweet orange hybrid [29]. The different parentages produced significant differences in aroma volatile composition and content, and sesquiterpene was the most important contributor. A total of 13 sesquiterpenes were identified in ‘Fortune’, however, only one sesquiterpene was found in ‘Murcott’ in 2013. The detection of fewer sesquiterpenes in ‘Murcott’ relative to other mandarin selections was reported, where four sesquiterpenes were identified in ‘Murcott’, compared to eight, 19, 23 and 20 in ‘Fallglo’, ‘Temple’, ‘Sanguinelli’ and ‘Ortanique’ mandarins [13, 16]. Compared to ‘Fortune’, ‘Murcott’ produced a similar number of volatiles, but with higher concentrations. This agrees with the previous report that ‘Murcott’ juice samples contained a higher level of many water-soluble volatiles compared to other mandarins, especially alcohols with one to five carbons [27].

### Mandarin juice aroma volatile correlations and segregation

Volatile compounds from the same biochemical pathway were normally clustered together, which indicated a co-regulation of these chemical compounds. In agreement with a study in strawberry [30], most of the detected volatile compounds exhibited positive correlations. The correlations are consistent with the existence of clustering of QTLs for volatile compounds from the same chemical family and few QTL intervals controlling the content of most of the detected volatile compounds. Clustering of QTLs for volatiles with the same structures was observed in apple [31], tomato [32], almond [33], strawberry [30], peach [34] and *Arabidopsis* [35]. The observed QTL clusters may correspond to a tight linkage of different loci or pleiotropic effects of a single locus on several volatiles. A single locus could affect an entire pathway by either encoding transcription factors to regulate genes coordinately or encoding enzymes catalyzing limiting steps in single pathways.

Consistent correlations were found between volatile content and fruit color in the ‘Fortune’ x ‘Murcott’ hybrids. Juice color space value *a* negatively correlated with monoterpene content over four harvests, while juice color space value *b* displayed relatively strong positive correlations with alcohol, aldehyde, ester, aromatic hydrocarbon and monoterpene in H1–13. Strong associations between color and aroma volatiles were observed in watermelon and tomato [36], and the relationship is likely to be a consequence of the degradation of carotenoids into volatile compounds. The norisoprene and monoterpene volatile compounds were influenced by carotenoid pigmentation, and the volatile geranial was apparently derived from the carotenoid lycopene [37]. Carotenoid contents in tomato fruit were associated directly with the emissions of carotenoid derived volatiles [38]. Higher production of apocarotenoid volatiles and lower concentration of carotenoids were found in ‘Temple’ than ‘Murcott’ fruits [16]. In our study, three of the six carotenoid derived volatile compounds, β-cyclocitral, neryl acetate, and geranyl acetone, consistently correlated with flavedo and juice color space value *a* and *a*/*b* ratio.

Some volatiles displayed qualitative variations and matched single locus inheritance. For example, valencene was present in only one parental line, ‘Fortune’, and in about 50% of the progeny, which matched the 1:1 ratio in 2013. In addition, a large-effect QTL was identified for valencene, with R^2^ being up to 59.82%. In strawberry, mesifurane and γ-decalactone were mapped as single Mendelian traits [30]. Spiller et al. [39] mapped nerol and neryl acetate as single Mendelian traits and found that geranyl acetate was controlled by two independent loci in diploid roses. It was observed in grape [40] and kiwifruit [41] that concentration of different terpenes was controlled by major genes. Transgression, which results in progeny from new combinations of multiple genes with more positive or more negative effects on a quantitative trait than were present in either parent, is often observed in the progeny derived from interspecific mating [42]. The wide variation in each volatile compound among the mandarin hybrids could be exploited to generate recombinants with markedly improved mandarin aroma flavor. Most of the detected compounds in mandarin F_1_ hybrids displayed transgressive segregations, and such strong transgressive segregation for volatile compounds was also observed in apple [31], peach [34], strawberry [30, 43] and tomato [32].

Terpenoids are the largest class of secondary metabolites in plants and play important roles in both plant and human health. In plants, isopentenyl diphosphate (IPP) and dimethylallyl diphosphate (DMAPP), the two C5 building blocks for all terpenoids, are derived from two independent pathways, the methylerythritol phosphate (MEP) pathway localized in the plastids [44] and the mevalonate (MVA) pathway localized in the cytosol [45]. The MEP pathway provides IPP and DMAPP for production of hemiterpene, monoterpene, and diterpene, whereas the MVA pathway provides IPP and DMAPP for sesquiterpene biosynthesis. In our study, 18 out of 27 sesquiterpenes detected in this study were grouped into cluster C based on correlation data, and the 41 QTLs identified for 18 sesquitepenes were mapped to four genomic regions. The overlapping of QTLs and strong correlations among sesquiterpenes suggested that in this mapping population the concentrations of several sesquiterpenes were controlled by a few distinct loci with pleiotropic effects, in accordance with the fact that all sesquiterpenes originate from similar biosynthetic pathways. Genes either affect the early stages of the MVA pathway or the early steps of terpene biosynthesis to have a strong effect on the numerous sesquiterpenes, or else they affect later steps in the terpene synthetic pathway to influence relatively few terpenes. Here, 11 out of 15 monoterpenes detected were grouped into cluster B based on their correlation coefficients. The 50 QTLs identified for 14 monoterpenes mapped to five genomic regions. The overlapping of QTLs and strong correlations among monoterpenes indicated pleiotropic effects of a few distinct loci. The monoterpenes and sesquiterpenes are believed to originate from quite different pathways, however, cross talk between the MEP and MVA pathways was reported in *Arabidopsis* [46]. In snapdragon flowers, only the plastid-localized MEP pathway was active in producing terpene compounds, providing IPP for both synthesis of monoterpenes in plastid and sesquiterpenes in cytosol [47]. In our study, total content of sesquiterpenes was positively correlated with total content of monoterpenes consistently over four harvests. The overlap of QTL clusters on FOR1.1, FOR3.4, MUR1.2 and MUR2 for both monoterpenes and sesquiterpenes may indicate tight linkage of distinct loci from MEP and MVA pathways, or the presence of major regulators in those loci, or a few genes affecting the early steps in one of the two pathways further influencing concentration of both monoterpenes and sesquiterpenes.

Esters are formed by esterification of alcohols and acetyl-CoA. Clustering of esters and alcohols is a common phenomenon. (E)-carveol and (E)-carvyl acetate were grouped into cluster B based on correlation. QTLs for ethanol and ethyl acetate were overlapped on FOR7.1. Hexanal and (E)-2-hexenal, formed from linoleic and linolenic acid respectively, and esters hexyl acetate and (Z)-3-hexenyl acetate were clustered into cluster D2. The aldehydes (E)-2-nonenal, (E)-2-decenal were grouped into cluster A, which was in agreement with overlapping of QTLs for (E)-2-nonenal, (E)-2-decenal and (E)-2-hexenal on MUR9. Decanal and decyl acetate were highly correlated in cluster B1, and QTLs for them overlapped on FOR1.1.

### Candidate genes for some QTLs

In this study, 12.14% of the QTLs for volatile compounds were stable over two or more harvests, which was less than that of aroma studies in peach [34] and strawberry [30], where 67.44 and 50% of QTLs were reproducible over two or more seasons, respectively. All genes related to the production of corresponding volatiles were considered and added to the candidate screening process, and four candidates (GPS1, TPS3, TPS4 and TPS14) were eventually identified in the corresponding map regions. The terpenoids are the largest and most diverse class of plant secondary metabolites with many aroma volatiles, including hemiterpenes (C5), monoterpenes (C10), sesquiterpenes (C15), homoterpenes (C11 and C16) and diterpenes (C20) [48]. In our mandarin aroma study, we detected 27 sesquiterpenes and 15 monoterpenes, some of them presented in more than 90% of all F_1_ hybrids. These could be considered as consensus volatile compounds, such as α-pinene, myrcene, limonene, α-phellandrene, α-terpinene, p-cymene, β-phellandrene, terpinolene and p-mentha-2,4(8)-diene. A total of 41 and 50 QTLs were identified for 18 sesquitepenes and 14 monoterpenes respectively. A QTL interval on MUR2 was identified for one alcohol, one aldehyde, five esters, five sesquiterpenes, nine monoterpenes and one unknown compound. The QTL interval corresponded to a single genomic region that contains genes *GPS1*, *TPS3*, *TPS4* and *TPS14*, encoding geranyl diphosphate synthase 1 (GPS1), terpene synthase 3 (TPS3), terpene synthase 4 (TPS4) and terpene synthase 14 (TPS14) respectively. GPS is responsible for generating the C10 terpene precursor geranyl pyrophosphate (GPP) by joining one IPP and DMAPP unit in the MEP pathway. Monoterpenes and sesquiterpenes are formed from terpene precursors GPP and farnesyl diphosphate (FPP) respectively catalyzed by the terpene synthases [49]. A total of 49 putative members of the terpene synthase family were reported in *Citrus* and grouped into six subfamilies [50]. GPP was converted to monoterpene (E)-β-ocimene catalyzed by a TPS3-encoded recombinant enzyme in *Arabidopsis* [51]. The allelic variation of terpene synthase genes, TPS2 and TPS3, produced the different level of emissions of (E)-β-ocimene and (E,E)-α-farnesene between two *Arabidopsis* accessions ‘Wassilewskija’ and ‘Columbia’ [52]. In *Arabidopsis*, caterpillar herbivory (*Pieris rapae*) induced the expression of the β-ocimene synthase TPS3 with increased myrcene emission [53]. TPS3 was responsible for the formation of camphene and tricyclene in tomato [54]. The monoterpene synthase TPS4 catalyzed the formation of mostly β-phellandrene from GPP in tomato [55]. The gene *TPS14* expressed in *Arabidopsis* flowers and encoded enzymes producing linalool [56]. TPS14 catalyzed the production of several bisabolene isomers and nerolidol from FPP in vitro [54]. GPS activity was reported in tissues producing abundant quantities of monoterpenes in plant species sage (*Salvia officinalis*) [57], *Pelargonium roseum* [58], *Vitis vinifera* L. cv Muscat de Frontignan [59], and *Abies grandis* [60]. GPS existed as a heterodimer with small and large subunits in *Antirrhinum majus* flowers, GPS small subunit mRNA expression level and protein were correlated with monoterpene biosynthesis [61].

## Conclusions

The aroma volatiles were investigated in mandarin parents ‘Fortune’, ‘Murcott’ and their derived F_1_ individuals using GCMS in 2012 and 2013 harvest seasons. A total of 148 volatiles were identified by the mass spectrometry, and among them, 28, 18 and 66 unique volatile compounds were detected in ‘Fortune’, ‘Murcott’ and the F_1_ hybrids respectively in 2013. Sesquiterpenes were the most important contributors to the difference between ‘Fortune’ and ‘Murcott’, as 13 sesquiterpenes were identified in ‘Fortune’ while only one sesquiterpene was found in ‘Murcott’ in 2013. A total of 206 QTLs were identified for 94 volatile compounds including 17 aroma active compounds, among them, 25 (12.14%) were consistent over two or more harvests. On MUR2, a QTL interval controlling multiple monoterpenes and sesquiterpenes corresponded to a genomic region that contains genes encoding geranyl diphosphate synthase 1 (GPS1), terpene synthase 3 (TPS3), terpene synthase 4 (TPS4) and terpene synthase 14 (TPS14). All 206 QTLs were validated in 13 citrus selections in 2014 harvest season, and validations on broader breeding germplasm and segregating populations are in progress, with some of them showing the potential for MAS in citrus breeding programs (data not shown). These SNPs could be used to screen mandarin individuals for some specific volatile compounds or even aroma active compounds after further validation. However, little is known regarding the contribution of individual volatile compounds, or about interactions between volatile compounds and the juice matrix, and other volatile compounds to the whole mandarin sensory profile. Further research is needed to understand which volatile compounds constitute the ‘desirable’ and ‘undesirable’ aroma for most of the consumers. The volatile compound composition should be correlated with the mandarin sensory profile generated by trained taste panels and consumer surveys, in order to understand the possible role of each individual volatile compound in the matrix for the mandarin sensory experience. The genetic linkage map should be combined with the mandarin sensory profile from trained panel and consumer surveys, in order to identify any QTLs linked to different mandarin sensory descriptors, and ‘desirable’ or ‘undesirable’ aroma volatiles for consumers. Once the QTLs are identified for the main mandarin sensory contributors, and for the ‘desirable’ or ‘undesirable’ aroma volatiles to most consumers, the molecular marker-assisted approach for mandarin fruit quality improvement may be utilized.

## Additional files

Additional file 1: Frequency of detection of the 148 detected juice volatiles in ‘Fortune’, ‘Murcott’ and the F1 progeny. This file contains a table showing the detection frequency of juice volatiles in ‘Fortune’, ‘Murcott’ and the F1 progeny over four harvests. (XLSX 19 kb)

Additional file 2: Relative content of the 148 detected juice volatiles in ‘Fortune’, ‘Murcott’ and the F_1_ progeny in H1–12, H2–12 and H2–13. This file contains a table showing the volatiles detected in juice samples from ‘Fortune’, ‘Murcott’ and the F_1_ progeny over three harvests. (XLSX 27 kb)

Additional file 3: Frequency distributions of relative content and number of juice volatiles in ‘Fortune’ x ‘Murcott’ in H1–12, H2–12 and H2–13. The value of relative content for each F_1_ hybrid was Log_10_ transformed, and then imported for analysis of distribution. A) Relative content of volatiles in H1–12. B) Number of volatiles in H1–12. C) Relative content of volatiles in H2–12. D) Number of volatiles in H2–12. E) Relative content of volatiles in H2–13. F) Number of volatiles in H2–13. (PPTX 52 kb)

Additional file 4: Correlation coefficients between chemical classes of volatiles determined in ‘Fortune’, ‘Murcott’ and the F_1_ progeny. This file contains a table showing the correlations between chemical classes of volatiles in the ‘Fortune’ x ‘Murcott’ population over four harvests. (XLSX 12 kb)

Additional file 5: Correlation coefficients between volatile groups and other physical and chemical attributes of ‘Fortune’, ‘Murcott’ and the F_1_ progeny fruits. This file contains a table showing the correlations between chemical classes of volatiles and fruit characteristics in the ‘Fortune’ x ‘Murcott’ population over four harvests. (XLSX 13 kb)

Additional file 6: Correlation coefficients between carotenoid derived volatiles and fruit color parameters in ‘Fortune’, ‘Murcott’ and the F_1_ progeny. This file contains a table showing the correlations between volatiles derived from carotenoids and colors in the ‘Fortune’ x ‘Murcott’ population over four harvests. (XLSX 10 kb)

Additional file 7: HCA and heat map representation of pairwise correlations between volatiles detected in ‘Fortune’ × ‘Murcott’ in H1–13. The blue-green-red scale bar represents low to high pair-wise correlation level. Clusters are indicated by different letters. (PPTX 708 kb)

Additional file 8: QTLs detected for juice volatiles in the mandarin F_1_ population, ‘Fortune’ x ‘Murcott’, using Kruskal-Wallis (K-W) and Composite Interval Mapping (CIM). This file contains a table showing the QTLs identified for juice volatiles in the ‘Fortune’ x ‘Murcott’ population over four harvests. (XLSX 29 kb)

Additional file 9: Gene in MUR2 that may participate in aroma volatiles regulation. This file contains a table showing the candidate genes from MUR2 that may regulate aroma volatiles production. (XLSX 8 kb)

## Abbreviations

ANOVA: Analysis of variance
CIM: Composite interval mapping
DMAPP: Dimethylallyl diphosphate
EM: Expectation maximization
FD: Fruit diameter
FOR: Fortune
FPP: Farnesyl diphosphate
FW: Fruit weight
GCMS: Gas chromatography mass spectrometry
GC-O: Gas chromatography-olfactometry
GPP: Geranyl pyrophosphate
GPS: Geranyl diphosphate synthase
H1–12: First harvest in 2012
H1–13: First harvest in 2013
H2–12: Second harvest in 2012
H2–13: Second harvest in 2013
HCA: Hierarchical cluster analysis
IPP: Isopentenyl diphosphate
JP: Juice percentage
LOD: Logarithm (base) of odds
MAS: Marker-assisted selection
MEP: Methylerythritol phosphate
MUR: Murcott
MVA: Mevalonate
PCA: Principal component analysis
QTL: Quantitative trait locus; LG: Linkage group
SNP: Single nucleotide polymorphism
SSC: Soluble solids content
TA: Titratable acidity
TPS: Terpene synthase
