# Supplementary figures and images for: Identification of QTLs controlling aroma volatiles using a ‘Fortune’ x ‘Murcott’ (Citrus reticulata) population
Source: BMC Genomics. 2017 Aug 22;18:646. doi: 10.1186/s12864-017-4043-5 (PMC5568196; doi:10.1186/s12864-017-4043-5)

## Slide 1
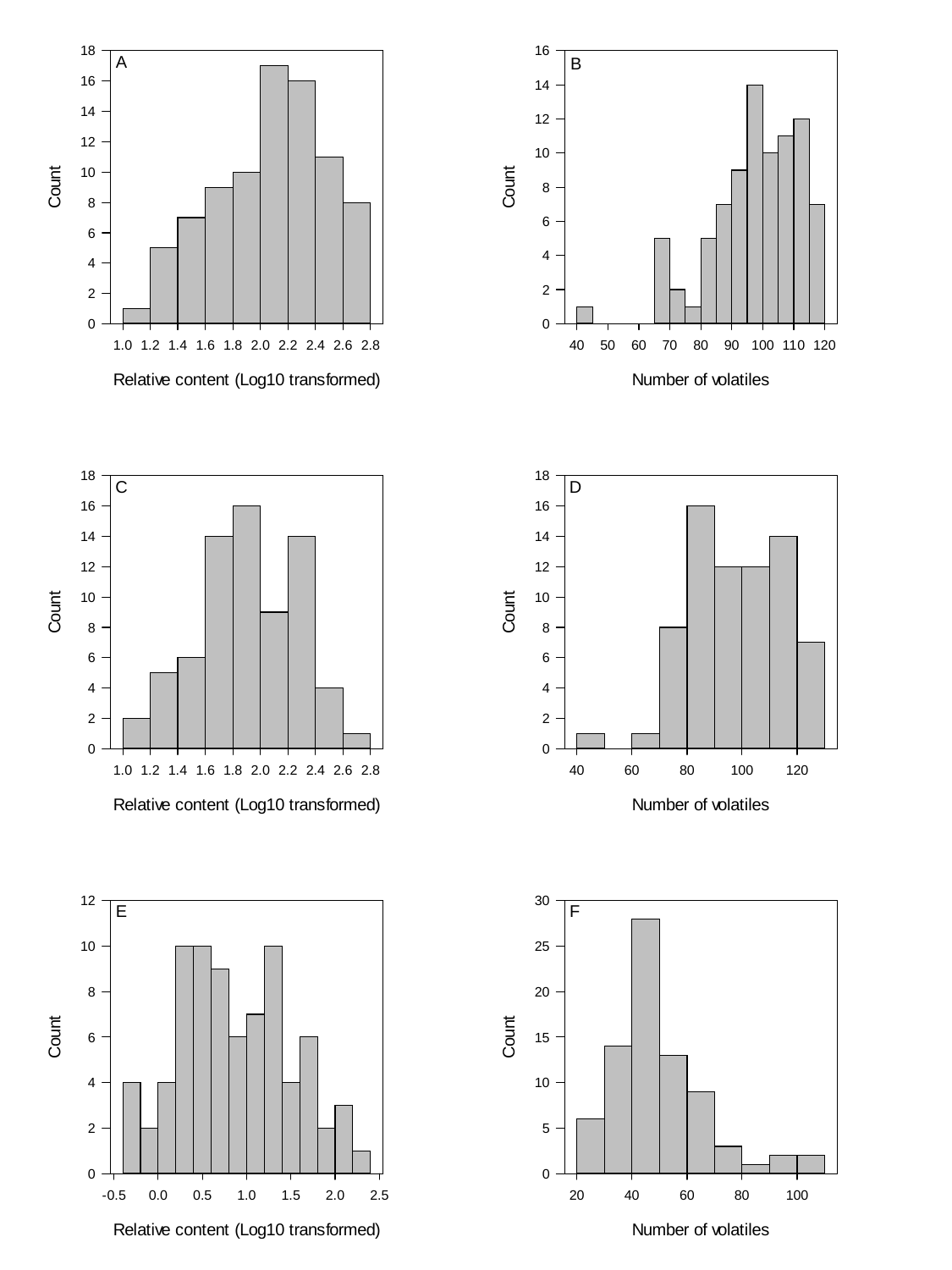

Supplement: Supplementary file 3 — Frequency distributions of relative content and number of juice volatiles in ‘Fortune’ x ‘Murcott’ in H1–12, H2–12 and H2–13. The value of relative content for each F1 hybrid was Log10 transformed, and then imported for analysis of distribution. A) Relative content of volatiles in H1–12. B) Number of volatiles in H1–12. C) Relative content of volatiles in H2–12. D) Number of volatiles in H2–12. E) Relative content of volatiles in H2–13. F) Number of volatiles in H2–13. (PPTX 52 kb) [file 12864_2017_4043_MOESM3_ESM.pptx]

## Slide 1
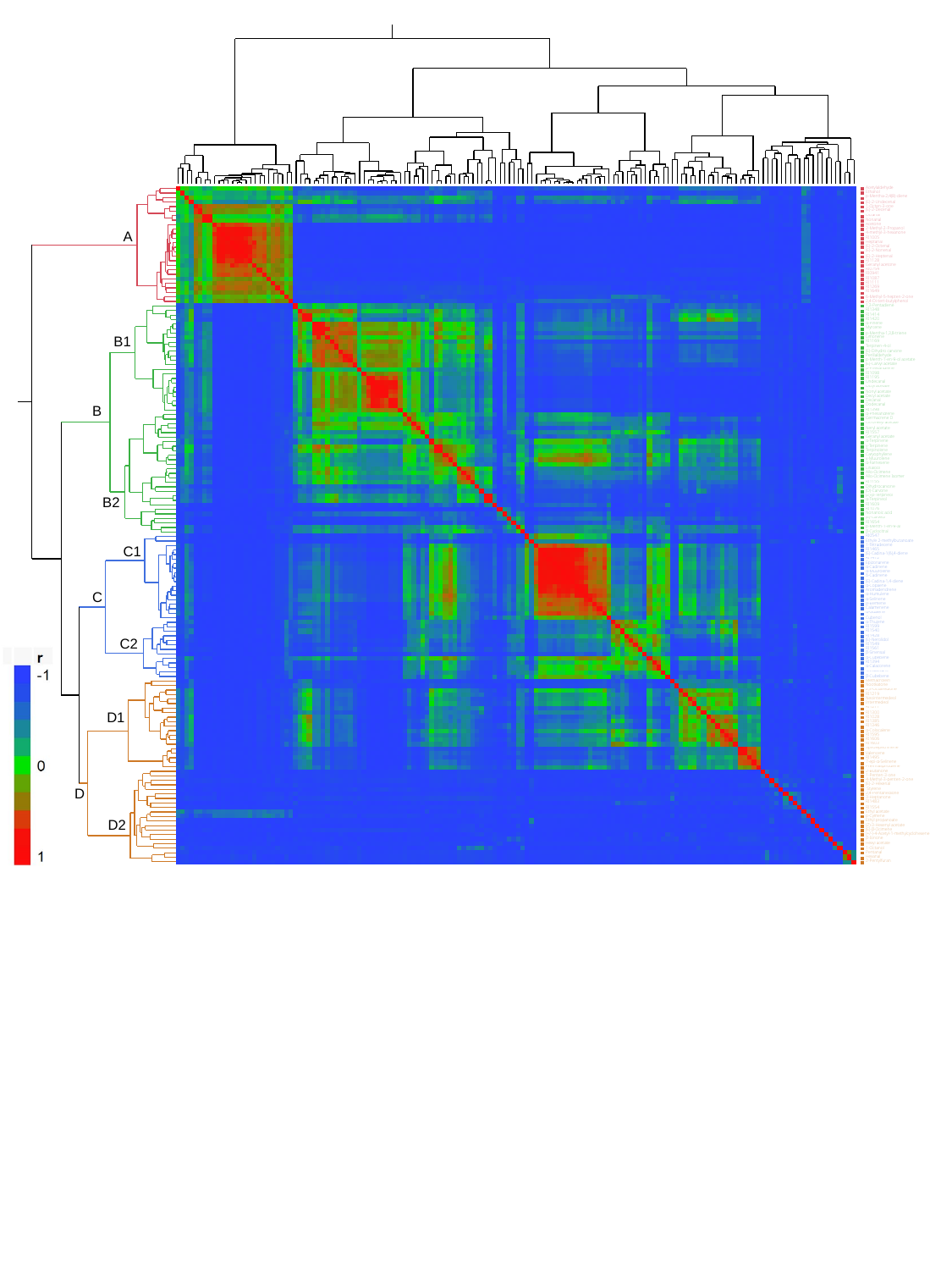

A
B1
B
B2
C1
C
C2
D1
D
D2

Supplement: Supplementary file 7 — HCA and heat map representation of pairwise correlations between volatiles detected in ‘Fortune’ × ‘Murcott’ in H1–13. The blue-green-red scale bar represents low to high pair-wise correlation level. Clusters are indicated by different letters. (PPTX 708 kb) [file 12864_2017_4043_MOESM7_ESM.pptx]
